# Determining the acceptance of e-mental health interventions in elite athletes using the unified theory of acceptance and use of technology

**DOI:** 10.3389/fspor.2024.1416045

**Published:** 2024-10-01

**Authors:** Sheila Geiger, Julia Aufderlandwehr, Anna Julia Esser, Theresa Schadendorf, Thomas Muehlbauer, Eva-Maria Skoda, Martin Teufel, Alexander Bäuerle

**Affiliations:** ^1^ Clinic for Psychosomatic Medicine and Psychotherapy, LVR-University Hospital Essen, University of Duisburg-Essen, Essen, Germany; ^2^ Center for Translational Neuro- and Behavioral Sciences (C-TNBS), University of Duisburg-Essen, Essen, Germany; ^3^ Division of Movement and Training Sciences/Biomechanics of Sport, University of Duisburg-Essen, Essen, Germany

**Keywords:** eHealth, UTAUT, sports, internet, survey

## Abstract

**Background:**

Elite athletes are exposed to many different sport-specific stressors that may put them at particular risk for mental health symptoms and disorders. E-mental health interventions could be a feasible option to support elite athletes in need. The aim of the present study was to assess the acceptance of e-mental health interventions among elite athletes and explore its underlying drivers and barriers.

**Materials and methods:**

A cross-sectional study was conducted with *N* = 382 elite athletes. Of these, *N* = 275 (71.99%, 167 females) were included in the statistical analyses. The impact of various sociodemographic, sport-related and medical characteristics on acceptance was assessed. EHealth-related data and acceptance of e-mental health interventions were examined using a modified assessment based on the Unified Theory of Acceptance and Use of Technology (UTAUT).

**Results:**

Overall, the acceptance of e-mental health interventions in elite athletes can be classified as high (*M* = 3.69 *SD* = 0.97). In the UTAUT regression model sex, financial situation, depression symptoms, digital confidence, digital overload as well as the UTAUT predictors performance expectancy, effort expectancy, and social influence predicted acceptance significantly.

**Conclusions:**

The UTAUT model has proven to be a valuable instrument in predicting of acceptance of e-mental health interventions in elite athletes. Given the strong association between acceptance and future use, new interventions should focus on the explored factors to establish effective e-mental health interventions for elite athletes.

## 1 Introduction

Participation in elite sports requires rigorous training, physical fitness, and mental toughness. Alongside these demanding requirements, elite athletes often experience significant psychological distress, including anxiety, depression, and sleep disturbance (1). Sport-specific stressors, such as high levels of competition, injury, and pressure to perform might influence an elite athlete's career (2). According to a systematic review and meta-analysis (1) the prevalence of mental health symptoms and disorders may be slightly higher among current and former elite athletes when compared to the general population. Specifically, the prevalence ranged from 19% for alcohol misuse to 34% for anxiety and depression among active elite athletes, and from 16% for distress to 26% for anxiety and depression among former elite athletes (1). This variance reflects the different types of mental health challenges faced by athletes, emphasizing the need for effective psychological support to address these issues comprehensively. Moreover, the heterogeneity of existing studies highlights the need for further high-quality research in this field to better understand and address the mental health needs of elite athletes.

However, elite athletes, despite experiencing risk factors and mental health symptoms, are less inclined to seek professional help compared to the general population (3). This reluctance can be attributed to factors like fear of stigmatization, potential impact on performance and career, the perception of seeking help as a sign of weakness, and a lack of understanding of mental health (4, 5). Apart from the effects of stigma, there could be additional obstacles that contribute to the challenges of receiving psychotherapeutic treatment. These barriers encompass individual factors like limited awareness of treatment choices or motivational issues arising from extended waiting periods to start treatment. Structural barriers, such as inadequate cross-sector coordination, limited availability of care options in specific regions, and prolonged waiting periods for psychotherapy appointments, also contribute to the issue (6).

To overcome these barriers and improve access to mental health care, innovative e-mental health solutions have emerged as a promising strategy. The term “eHealth” broadly encompasses the use of electronic devices such as cell phones and computers to enhance medical care (7). EHealth interventions can be divided between self-help methods, computer-assisted treatments, and telehealth services. Self-help methods involve self-guided efforts to cope with (mental) health issues, often through self-help books or online programs (8, 9). Computer-assisted treatments use computer programs to deliver aspects of psychotherapy directly to patients (10, 11). Telehealth services, as defined by the WHO (12), involve the delivery of health care services where patients and providers are separated by distance, using information and communication technology for diagnosis, treatment, and consultation. E-mental health interventions, i.e., the provision of mental health services via digital technologies, offer flexible, cost-effective, and accessible support, overcoming structural barriers and reaching a larger number of individuals compared to traditional face-to-face counselling (13, 14). Multiple studies investigating the efficacy of e-mental health interventions in treating various mental disorders and health-related outcomes have demonstrated outcomes similar to those achieved through in-person therapy (14–17). This study focuses on e-mental health interventions that fall under the category of telehealth.

Thus, e-mental health interventions have proven to be useful, but are not yet integrated into everyday life, as the utilization of specific e-mental interventions continues to be minimal (18–20). A key consideration regarding technology use is understanding individuals’ motivations for adopting it. The most important factor in this regard is the degree to which a technology is accepted. Previous studies show that the acceptance of e-mental health interventions is generally rather low (21–23) whereasore recent studies suggest that the acceptance of e-mental health interventions is generally rather moderate to high (24–28).

Hence, assessing the acceptance of e-mental health interventions as well as influencing factors among elite athletes is crucial. Until now, no research has been conducted using validated measurement methods to evaluate the acceptance of e-mental health interventions and its predictors among elite athletes. Consequently, the current study employs the Unified Theory of Acceptance and Use of Technology [UTAUT; (29)]. The UTAUT has been found to be a valuable framework for evaluating the acceptance of eHealth-interventions in a number of studies (22, 23, 30, 31). The UTAUT model identifies three key factors that contribute to an individual's acceptance (or behavioral intention) of any technology: performance expectancy, effort expectancy, and social influence. Performance expectancy refers to the extent to which a person believes they will benefit from using the technology, while effort expectancy measures the ease of use associated with the technology, and social influence reflects the degree to which important others, such as family or friends, believe the person should use the technology.

Based on previous research that has explored acceptance in other populations, this study aims to fill the gap by setting forth the following objectives: The first objective is to assess the level of acceptance of e-mental health interventions in elite athletes. The second objective is to evaluate whether acceptance levels of e-mental health interventions vary among elite athletes based on sociodemographic, eHealth, or sport-related factors. The third objective is to explore the factors that influence the acceptance of e-mental health interventions among elite athletes.

## 2 Materials and methods

### 2.1 Study design and participants

The study was carried out as a web-based, cross-sectional survey among German adult (aged ≥18 years) elite athletes from December 2021 to December 2022. The criteria for elite athletes were determined, in part, by the International Olympic Committee's consensus statement and included (1) alignment of life to sports, (2) striving for excellence, and (3) participation in professional or Olympic competitions (32–34). Participants were recruited through regional and national sport clubs and associations, social media and coaches. The participation was anonymous and voluntary and there was no financial or material incentive offered. Furthermore, before starting the survey, electronic informed consent was obtained. The setup of the study was developed in accordance with the Declaration of Helsinki and approved by the Ethics Committee of the Faculty of Medicine of the University of Duisburg-Essen (19-8947-BO). The survey was answered by 382 participants, but 29 (7.59%) of them were under 18 years old and 78 (20.42%) did not meet the criteria of elite athletes, resulting in their exclusion at the start of the survey. Therefore, the final sample size for data analysis comprised 275 participants.

### 2.2 Measures

#### 2.2.1 Sociodemographic and sport-related data

In this study, sociodemographic data was collected through self-reported measures, including chronological age, sex, level of education and living as well as family and employment status. Financial situation was assessed on a 10-point-Likert scale (0 = “I barely get by”, 10 = “I don't have to restrict myself in any way”). Moreover, sport-related variables, including type of sport, years in elite sport, days at home during the last month as well as earning a living through sport, were assessed.

#### 2.2.2 Psychometric data

The Patient Health Questionnaire-8 (PHQ-8) was used to assess depression symptoms (35). This questionnaire comprises eight items, and participants responded using a four-point Likert scale (0 = never to 3 = almost every day). The internal consistency of the PHQ-8 in this study was found to be high (Cronbach's *α* = 0.85). The Generalized Anxiety Disorder Scale-7 (GAD-7) is a validated (36) instrument to assess symptom severity and probability of GAD. It contains seven items, each scored from 0 to 3, indicating the frequency of symptoms experienced over the past 2 weeks. A score of 0 means the symptom has not been experienced at all while a score of 3 stands for a daily occurrence of the symptom. Cronbach's *α* in our study was high (*α* = 0.85).

#### 2.2.3 eHealth-related data

To assess eHealth-related data, participants rated three items regarding their digital confidence (use of digital media, online platforms, and digital devices) on a five-point Likert scale (1 = not confident at all, 5 = very confident). The internal consistency of this scale was found to be excellent (Cronbach's *α* = 0.90). Moreover, three self-generated items were utilized to measure internet anxiety, which is defined as the fear or apprehension that individuals experience when using the internet (37). Participants provided responses on a five-point Likert scale (e.g., “I have concerns about using the internet”, 1 = does not apply to me, 5 = does apply to me). The internal consistency of this scale was found to be high (Cronbach's *α* = 0.80). Additionally, we used three self-generated items with a five-point Likert scale to assess digital overload, defined as the stress and burden felt due to the constant accessibility and constant use of digital devices (e.g., “I feel burdened by the constant accessibility via cell phone or mail”, 1 = does not apply to me, 5 = does apply to me). Cronbach's *α* was sufficient [*α* = 0.73; (38)]. All scales used in the study have been well-established through their application in prior research studies (24–26, 39).

#### 2.2.4 Acceptance and UTAUT predictors

To measure the acceptance towards e-mental health interventions a modified questionnaire based on the UTAUT was applied (40). The modified UTAUT questionnaire comprises 14 items that are rated on a five-point Likert scale (0 = strongly disagree, 4 = strongly agree). The dependent variable in this study is acceptance, operationalized as behavioural intention (e.g., “I would like to try a psychological online intervention”), which was assessed using four items. The internal consistency of acceptance (behavioural intention) was found to be excellent (Cronbach's *α* = 0.90). The two UTAUT predictors, effort expectancy (e.g., “Using a psychological online intervention would not be an additional burden for me”) and social influence (e.g., “My coach would approve the use of a psychological online intervention”), were each measured using three items and demonstrated the following internal consistencies: Cronbach's *α* = 0.63 for effort expectancy and *α* = 0.85 for social influence. While the Cronbach's *α* for effort expectancy is relatively low, which may indicate questionable internal consistency, this finding contrasts with previous studies where the scale has shown adequate reliability (31). Performance expectancy (e.g., “A psychological online intervention could help me improve my mental health”) was assessed using four items and exhibited excellent internal consistency (Cronbach's *α* = 0.91).

### 2.3 Statistical analysis

The statistical analyses were performed using version 26 of SPSS Statistics by IBM in New York, NY, USA and RStudio version 4.0.2 by RStudio PBC in Boston, MA, USA. Firstly, the internal consistencies of various psychometric questionnaires were determined, and descriptive statistics were calculated. Additionally, in accordance with prior research, acceptance (BI) was classified into low (1.00–2.34), moderate (2.35–3.67), and high (3.68–5.00) categories (23, 26–28). The sum scores for the GAD-7 and PHQ-8 scales were computed. Mean acceptance (BI) differences between groups based on sociodemographic, sport- and eHealth-related data were examined using *t*-tests and ANOVAs, with the latter being used for variables with multiple categories. The level of significance was established at *α* = 0.05 (two-sided test). *Post-hoc* tests were conducted following the mean comparisons, and *α* correction was implemented using the Bonferroni method. Considering the present sample size (*N* = 275), normal distribution in the variables was assumed [see central limit theorem; (41)]; hence, parametric tests were used. We conducted a hierarchical regression analysis, following approaches in comparable literature, to maintain consistency and comparability of results (23, 25, 26, 39, 40, 42). The blocks were structured as follows: (1) sociodemographic and sport-related variables to control for baseline characteristics, (2) mental health variables to account for psychological factors, (3) eHealth-related variables to capture digital engagement, and (4) UTAUT predictors to assess technology acceptance factors.

## 3 Results

### 3.1 Sample characteristics

The participants (*N* = 275; 167 females) were *M* = 23.65 years old (*SD* = 6.29), while three quarters of the sample were 18–25 years old. 42.5% of the athletes rated their financial situation as okay or better (*M* = 6.95; *SD* = 2.09). Regarding the type of sports, 133 participants were active in team sports, while 190 did individual sports and 47 athletes participated in both. On average, the athletes were active in elite sport for 9.69 years (*SD* = 5.25) and spent an average of 21.79 (*SD* = 7.42) days at home in the last month. The participants reported high digital confidence (*M* = 4.12; *SD* = 0.73) and low internet anxiety (*M* = 1.84; *SD* = 0.80), while their digital overload was moderate (*M* = 2.71; *SD* = 0.96). See Table 1 for a full description of the study population.

**Table 1:** Description of the study sample (*N* = 275).

Variable	*n*	%
Family status		
Kids (underage)	15 (12)	5.5 (4.4)
Single	187	68.0
Partnership	63	22.9
Married	21	7.6
Divorced	2	0.7
Widowed	0	0.0
Other	2	0.7
Living situation		
With parents	75	29.8
Alone	66	24.0
Flat sharing	58	21.1
With partner	49	17.8
With partner and child(ren)	12	4.4
Other	15	5.5
Education		
High school diploma	159	57.8
University degree	63	22.9
Secondary school degree	15	5.5
Secondary school degree	1	0.4
Vocational education	14	5.1
Ongoing school education	15	5.5
No degree	2	0.7
Other	6	2.2
Employment status		
Employed	48	22.9
Self-employed	15	7.1
Civil servant	11	5.2
Other	26	12.4
Types of sports		
Ball sports	90	32.8
Combat sports	19	6.9
Strengths sports	15	5.5
Track and field	27	9.9
Equestrian sports	17	6.2
Gymnastics	11	4.0
Dance sports	5	1.8
Water sports	81	29.6
Winter sports	2	0.7
Trend sports	7	2.6
Earn a living through sports		
Yes	53	19.3
No	222	80.7

Sample characteristics in absolute numbers (*n*) and in percent (*%*).

### 3.2 Research objective 1 and 2: acceptance of eHealth-interventions and influencing factors

The general acceptance of e-mental health interventions was high (*M* = 3.69 *SD* = 0.97). Regarding the degree of acceptance, the sample of 275 participants was categorized into groups as follows: 35 (12.7%) participants showed low, 65 (23.6%) showed moderate, and 175 (63.6%) showed high acceptance.

There was a significant difference in acceptance between female and male participants (*t*_189.07_ = −2.48; *p* = .014), with females showing higher acceptance than males. In addition, elite athletes in individual sports were found to have significantly higher acceptance scores compared to those who participate in team sports (*F*_2,272_ = 3.47; *p* = .032). However, acceptance did not differ between living situations. A report summarizing the results regarding differences in acceptance is presented in Supplementary Table S1.

### 3.3 Research objective 3: predictors of acceptance

In the multiple hierarchical regression analysis, it was shown that sociodemographic predictors explained 7.3% of the variance in acceptance in the first step (*R^2^* = 0.073; *F*_6,268_ = 3.512; *p* = 0.002). Of the sociodemographic predictors sex (*β* = 0.144; *p* = 0.017) and financial situation predicted acceptance significantly (*β* = −0.210; *p* < 0.001). In the second step, mental health variables explained another 6.8% of the variance in acceptance (Δ *R*² = 0.068; *F*_8,266_ = 5.453; *p* < 0.001). In detail, depression symptoms significantly predicted acceptance (*β* = 0.261; *p* = 0.013), whereas presence of generalized anxiety symptoms was no significant predictor of acceptance (*p* = 0.818). In the third step, eHealth-related variables were included and explained another 4.9% of variance (Δ *R*² = 0.049; *F*_11,263_ = 5.592; *p* = 0.002). Digital overload (*β* = 0.149; *p* = 0.024) and digital confidence (*β* = 0.145; *p* = 0.013) predicted acceptance significantly, whereas internet anxiety was no significant predictor of acceptance (*p* = 0.127). In the last step, UTAUT predictors explained 47.4% (Δ *R²* = 0474; *F*_14,260_ = 36.702; *p* < 0.001) of the variance resulting in a total explained variance in acceptance of 66.4%. Of the variables included in step four, performance expectancy (*β* = 0.477; *p* < 0.001), effort expectancy (*β* = 0.192; *p* < 0.001), and social influence (*β* = 0.203; *p* < 0.001) significantly predicted acceptance. Table 2 displays the parameters of the hierarchical regression model of acceptance.

**Table 2:** Hierarchical regression model of acceptance.

Predictor	*β*	B	*T*	*R*²	Δ *R*²	*p-*value
Step 1: sociodemographic and sport-related variables				.073	.073	
Sex	.144	.286	2.403			.017
Age	.064	.010	.945			.345
Financial situation	−.210	−.098	−3.466			<.001
Days at home	.051	.007	.851			.395
Years in elite sports	.037	.007	.560			.576
Earn a living through sports	−.009	−.022	−.149			.882
Step 2: psychometric variables				.141	.068	
PHQ8_sum	.261	.055	2.506			.013
GAD7_sum	.024	.006	.230			.818
Step 3: eHealth-related variables				.190	.049	
Digital confidence	.145	.194	2.488			.013
Internet anxiety	.100	.123	1.531			.127
Digital overload	.149	.151	2.266			.024
Step 4: UTAUT predictors				.664	.474	
UTAUT_PE	.477	.523	9.059			<.001
UTAUT_EE	.192	.254	3.934			<.001
UTAUT_SI	.203	.231	4.182			<.001

*N* = 275. In Step 2, 3 and 4, only the newly included variables are presented. *Β*, standardized coefficient beta; B, unstandardized coefficient beta; *R*², determination coefficient; Δ *R*², changes in *R*²; PHQ8_sum, sum score of the patient health questionnaire-8; GAD7_sum, sum score of the generalized anxiety disorder scale-7; PE, performance expectancy; EE, effort expectancy; SI, social influence.

## 4 Discussion

### 4.1 Principal findings

E-mental health interventions are an effective alternative to personal therapy that combats stigma and ensures accessibility. Assessing the acceptance and predictors of these interventions is critical to future utilization and implementation. This study is the first to investigate this among elite athletes. General acceptance of e-mental health interventions among elite athletes was high, with 23.6% showing moderate and 63.6% showing high acceptance. Acceptance was associated with sex and being engaged in team or individual sports. In the multiple hierarchical regression analysis, sex, financial situation, depression symptoms, digital confidence, and digital overload were significant predictors of acceptance. Of the UTAUT predictors, performance expectancy, effort expectancy and social influence were significant predictors of acceptance and explained a high percentage of variance. The overall model provided 66.4% of explained variance in acceptance of e-mental health interventions.

Overall, acceptance levels in this study exceeded or were as high as those observed in previous studies similarly examining the acceptance of e-mental health interventions among various populations (24, 25–28, 39, 43), which can be a good prerequisite for the implementation and actual use of such interventions in elite sports. Especially for elite athletes who travel a lot, it can be beneficial to use treatment alternatives that are independent of location and time. An additional advantage is that the usage of mental health interventions can reduce fear of stigmatization. In line with this, Klein et al. (44) found that high levels of stigma were associated with a preference for e-mental health interventions.

### 4.2 Predictors of acceptance

Several predictors of acceptance have already been identified in various studies exploring the acceptance of e-mental health interventions among different samples. These predictors include sex (45, 46) and internet anxiety (47). The results of the present study also suggest that sex is a predictor for acceptance. Female athletes showed significantly higher acceptance than male athletes. There are studies that yield consistent results (24, 25), while others demonstrate no difference between sex (31, 48). The results can be explained by the observation that women have a more positive attitude towards seeking professional psychological help than their male counterparts (49). Moreover, our results align with research indicating that female elite athletes tend to report higher levels of psychological burden compared to their male counterparts (50, 51). Therefore, one could expect that psychologically burdened individuals would be particularly receptive to embracing new digital healthcare options. Interestingly, age did not emerge as a significant predictor in this study, which may be attributed to the relatively young average age of the sample, resulting in a more homogenous group with similar familiarity and comfort with digital technology. Also the sport-related variables (number of days spent at home, years in elite sports, and earning a living through sports) did not emerge as significant predictors of acceptance. This could be because these factors may not have a direct impact on the acceptance of e-mental health interventions. Athletes in different stages of their careers or with varying financial situations could experience similar needs and barriers regarding mental health interventions, leading to insufficient variation in these variables to produce significant results.

Digital confidence and digital overload emerged as further significant predictors of acceptance. Similarly, a potential explanation in this context could be that increased digital overload contributes to heightened psychological burden, consequently resulting in a greater willingness to accept e-mental health interventions. Moreover, individuals who are more confident in using digital media seem to accept them more. Conversely, individuals who lack digital confidence or have limited experience with digital media encounter significant barriers when accessing innovative e-mental health services. This may be because those more aware of their digital habits are also more receptive to solutions that can help manage their digital interactions. During the development of new e-mental health interventions, these varying capabilities should be taken into account. In contrast to previous research (47), internet anxiety was not found to be a significant predictor in this study. This might be because digital technologies have become increasingly commonplace, even in elite sports. The majority of athletes may possess sufficient digital competence, allowing them to overcome moderate internet anxiety when considering e-mental health interventions.

Another significant predictor of acceptance is the financial situation as assessed by the elite athletes. One possible reason for this observation could be that elite athletes who perceived their financial situation negatively experienced greater psychological burden. Consequently and in line with the other findings, they displayed a greater inclination towards accepting eHealth-interventions. Notably, the financial situation was found to be highly significant with a negative correlation coefficient, indicating that the worse the financial situation, the higher the acceptance of e-mental health interventions. This finding underscores the possibility that greater financial strain leads to increased psychological stress, thereby heightening acceptance. Consistent with this, depressive symptoms were also found to be an additional predictor of acceptance. Similar findings were also evident in other studies (24, 25, 46). In line with this, elite athletes showed significantly lower acceptance if they were engaged in team sports compared to individual sports. This fits with research showing that team athletes are less likely to suffer from anxiety or depression than individual athletes (52). Another plausible explanation could be that individual athletes spend significantly more time alone than team athletes and are therefore more receptive to e-mental-health interventions. Additionally, individual athletes may perceive higher levels of anonymity when using e-mental-health interventions, as there are fewer people who could potentially find out about their participation compared to those in team sports.

Regarding the UTAUT model, the results of this study provide strong support for the validity in measuring the acceptance of e-mental health interventions, supporting prior research outcomes (25, 53–56). Specifically, the key predictors, social influence, effort expectancy, and performance expectancy accounted for 66.4% of the variance in acceptance, demonstrating a high and comparable level of explained variance to the original UTAUT validation study (70%) conducted by Venkatesh, Morris (40). Moreover, performance expectancy emerged as the most important predictor of acceptance, with negative outcome expectations predicting lower intention to use, which is consistent with previous research findings (23, 25, 57, 58). Furthermore, this observation is consistent with literature indicating that performance expectancy is a predictor of treatment outcome in psychotherapy (59). The strong relationship between performance expectancy and the acceptance of e-mental health interventions (*β* 03D; 0.477; *p* < 0.001) emphasizes the necessity for transparent eHealth education that openly deals with misunderstandings or unrealistic expectations.

Social intention includes a person's belief that important people in his or her life, e.g., family or friends, would endorse the use of e-mental health interventions (40). This highlights the systemic dimension of person-environment interaction in the context of elite sport. Promoting acceptance can be facilitated by the positive attitudes of significant individuals toward e-mental health interventions and the willingness of physicians to recommend these programs. Specifically, general practitioners can play a vital role as key influencers in encouraging the adoption of eHealth solutions (60–62). A study by Van Voorhees, Hsiung (62) demonstrated that uptake of an e-mental health intervention increased when clinicians offered client-centered information which aimed to strengthen intrinsic motivation. Therefore, facilitation of acceptance needs to include relevant parties of health care (e.g., practitioners, clinicians, administrators) as important mediators of eHealth implementation.

In addition, it is critical to emphasize that consideration of the influencing factors of effort expectancy, performance expectancy, and social influence during the development and implementation phases is essential to improving acceptance. While these three key predictors are of utmost importance within the UTAUT model, our findings highlight the need to consider additional factors for a comprehensive understanding of acceptance and how to maximize it. Here, so-called acceptance facilitating interventions can be used to reduce people's fears and misconceptions about e-health interventions and thus increase acceptance (21, 22, 30).

The high acceptance of e-mental health interventions among elite athletes underscores their practical value. Coaches, sports staff, and medical professionals should integrate these tools into athletes’ routines and support their effective use. Addressing the identified significant predictors, such as digital confidence and depressive symptoms, is crucial when developing and implementing these interventions for this target group. Considering athletes’ travel schedules, it is essential that e-mental health solutions are accessible from any location. Emphasizing early detection, self-management skills, and support from key stakeholders can enhance the impact of these interventions (63). By implementing these strategies, e-mental health tools can more effectively address the mental health needs of elite athletes.

### 4.3 Limitations

Interpretation of our results should regard the following limitations. Firstly, our study design was a web-based cross-sectional study. The cross-sectional study design does not display any change over time and no statement to causality can be made. Longitudinal studies could address this limitation by tracking changes in acceptance and predictors over time. Because internet access was inevitable for participation, the sample of elite athletes who took part in our study might be more open minded about eHealth-interventions than the average elite athlete. As the sample was primarily made up of young people and the group of elite athletes tends to be younger and to have internet access, we do not really assume any bias here. Furthermore, it is likely that elite athletes who do not have much psychological support at this point in time are more interested in e-mental health interventions and show higher levels of acceptance than others. Another relevant factor might be that the study was conducted during different stages of the COVID-19 pandemic, a period marked by a rapid establishment and acceptance of digital patient care approaches (64). The pandemic highlighted the need for accessible psychological care as rates of depression, anxiety, and stress increased (65, 66). Additionally, high satisfaction with telemedicine during this time (67) suggests that the pandemic may have contributed to the observed higher acceptance of e-mental health interventions.

Since all data was self-reported, they are of limited reliability. For the evaluation of mental health, we used validated and established measures (GAD-7, PHQ-8), but it should be considered that all of these are symptom-based measures and therefore have a limited diagnostic accuracy. Another limitation is the low internal consistency of the effort expectancy scale (Cronbach's *α* = 0.63), which may affect its reliability. This was unexpected given its prior validation (31). Future research should revise or add items to improve the scale's reliability and ensure accurate measurement of effort expectancy in e-mental health interventions. As an operationalization of acceptance, we used behavioral intention. A prediction to actual use cannot be made because the intention to use does not always lead to actual use, known as the intention-behavior-gap (68). Further longitudinal research will be needed to assess how acceptance relates to actual behavior. We suggest measuring uptake rates and explore further barriers and incentives to use e-mental health interventions for elite athletes.

### 4.4 Conclusions

The results of this study support the assumption that elite athletes readily accept e-mental health interventions, providing a solid foundation for the integration of novel e-mental health interventions. Core predictors of acceptance included factors such as performance expectancy, effort expectancy, and social influence as well as depressive symptoms, digital confidence, and digital overload. Understanding these influencing factors is crucial for tailoring e-mental health interventions and encouraging their actual use in situations where in-person treatment is scarce, e-mental health interventions present a viable option. Stakeholders should consider the specific expectations, needs, and digital skills of elite athletes to enhance the effectiveness and adoption of these interventions. Future research should focus on longitudinal studies to explore how acceptance evolves over time and its impact on actual behavior. Additionally, improving the reliability of measurement tools and understanding the intention-behavior gap will strengthen the evidence base for e-mental health interventions. Finally, this study emphasizes the necessity of considering participants’ expectations, needs, and capabilities in the development and implementation of innovative treatment approaches. Addressing these aspects will contribute to better support for elite athletes and the optimization of mental health care in this population.

## Data Availability

The raw data supporting the conclusions of this article will be made available upon reasonable request to the corresponding author.
