# Supplementary material for: Determining the acceptance of e-mental health interventions in elite athletes using the unified theory of acceptance and use of technology
Source: Front Sports Act Living. 2024 Oct 1;6:1416045. doi: 10.3389/fspor.2024.1416045 (PMC11474182; doi:10.3389/fspor.2024.1416045)
Supplement: Supplementary file 1 [file Table1.docx]

Supplementary Material

**Determining the acceptance of e-mental health interventions in elite athletes**

**using the unified theory of acceptance and use of technology**

Sheila Geiger^1,2*^, Julia Aufderlandwehr^1,2^, Anna Julia Esser^1,2^, Theresa Schadendorf^1,2^, Thomas Muehlbauer^3^, Eva-Maria Skoda^1,2^, Martin Teufel^1,2^, Alexander Bäuerle^1,2^

*** Correspondence:** Sheila Geiger [sheila.geiger@uni-due.de](mailto:sheila.geiger@uni-due.de)

**Table S1.** Differences in acceptance (Unified Theory of Acceptance and Use of Technology behavioral intention scale) by sociodemographic, sport- and eHealth-related data.

| **Variable** | ***n*** | ***%*** | **Mean (*SD*)** | **Test** | ***p*-Value** |
| --- | --- | --- | --- | --- | --- |
| **Sex** |  |  |  | *t*_189_ = -2.48 | .014* |
| Female | 167 | 60.7 | 3.77 (0.86) |  |  |
| Male | 108 | 39.3 | 3.45 (1.11) |  |  |
| **Living situation** |  |  |  | *F*_7,267_ = 1.0 | .434 |
| With parents | 75 | 27.3 | 3.54 (1.08) |  |  |
| Alone | 66 | 24.0 | 3.82 (0.88) |  |  |
| Flat sharing | 58 | 21.1 | 3.84 (0.87) |  |  |
| With partner | 49 | 17.8 | 3.68 (0.99) |  |  |
| With partner and child(ren) | 12 | 4.4 | 3.75 (0.43) |  |  |
| Other | 15 | 5.5 | 3.3 (1.33) |  |  |
| **Individual/ Team sport** |  |  |  | *F*_2,272_ =3.47 3.47 | .032* |
| Individual sport | 143 | 52.0 | 3.84 (0.90) |  |  |
| Team sport | 85 | 30.9 | 3.51 (1.02) |  |  |
| Individual and team sport | 47 | 17.1 | 3.59 (1.07) |  |  |
| **Previous experience with  eHealth-Interventions**    N#  Interventions |  |  |  | *F*_2,272_ = 2.28 | .104 |
|  |  |  |  |  |  |
| Already perceived online support offers | 19 | 6.9 | 4.12 (0.69) |  |  |
| Knowledge about possibility of claiming  online support but not participated yet | 56 | 20.4 | 3.75 (0.91) |  |  |
|  |  |  |  |  |  |
| No knowledge about possibility of  claiming online support | 200 | 72.7 | 3.64 (1.01) |  |  |

Note: Total *N* = 275. **p* < 0.05.
